# Incidence and clinical features of kawasaki disease in Catalonia (Spain)

**DOI:** 10.1186/1546-0096-12-S1-P123

**Published:** 2014-09-17

**Authors:** Judith Sanchez-Manubens, Jordi Anton, Fredy Prada, Rosa Bou, Estibaliz Iglesias, Joan Calzada-Hernandez, Vicenç Torrente-Segarra, Samuel Hernandez, Silvia Ricart, Marc Tobeña, Anna Fernandez, Montserrat Gispert-Sauch, Aina Sanchez, Mariona Bonet, Neus Rius, Sonia Corral, Olga Calavia

**Affiliations:** 1 Rheumatology Unit, Department of Pediatrics, Hospital Parc Taulí, Sabadell, Spain; 2 Rheumatology Unit, Department of Pediatrics, Hospital Sant Joan de Déu, Esplugues de Llobregat, Spain; 3 Cardiology Department, Hospital Sant Joan de Déu, Esplugues de Llobregat, Spain; 4 Pediatrics Department, Hospital Vall d'Hebron, Barcelona, Spain; 5 Pediatrics Department, Hospital Arnau de VIlanova, Lleida, Spain; 6 Pediatrics Department, Hospital Josep Trueta, Girona, Spain; 7 Pediatrics Department, Consorci Sanu¡itari de Terrassa, Terrassa, Spain; 8 Pediatrics Department, Hospital del Mar, Barcelona, Spain; 9 Pediatrics Department, Hospital Sant Joan de Reus, Reus, Spain; 10 Pediatrics Department, Hospital General de Granollers, Granollers, Spain; 11 Pediatrics Department, Hospital Joan XIII, Tarragona, Spain

## Introduction

Kawasaki disease (KD) is an acute self-limited systemic vasculitis relatively common in childhood. In Japan, last published survey shows an incidence up to 239.6/10^5^ children <5 years old (yo). In Madrid (Spain) a retrospective study with no well defined reference area showed an incidence of 15.1/10^5^ children <5yo.

## Objectives

To ascertain the incidence and clinical features of KD in Catalonia, autonomous region in northeast Spain with 7.5 million inhabitants.

## Methods

Observational population-based study including all Catalan hospitals with Pediatric Units, both public and private management. Retrospective data retrieval was performed for the last 10 years (2004-2013). The presence of coronary aneurysms (CA) in echocardiology was based in the body surface area according to the American Heart Association.

## Results

Data from 399 patients from 33 different hospitals was analyzed. Of those, 233 (58.4%) patients had complete KD, 159 (39.8) incomplete KD and 7 (1.7%) were considered atypical KD. The mean annual incidence was 3.5/10^5^ children <14yo and 8/10^5^ children<5yo (mean age 37±33months(m), range 1.3-191.3m). KD was more frequent among boys (59.6%, p<0.01). Mean delay between onset of the disease and diagnostic was 7.2±5.3 days. Ethnic distribution was: Caucasian 279 patients(69.9%), North African 26 (6.5%), Amerindian 21 (5.2%), Asian 14 (3.5%) and Sub-Saharan 4 (1%). Ethnicity was not available in 55 (13.8%) patients. Distribution of classical manifestations for KD was: fever in 100% of patients, changes in extremities 40.3% (desquamation in 31% of them), exanthema 84.2%, conjunctival injection 79.7%, changes in lips and oral cavity 55.6% and lymphadenopathy 28.8%. Other clinical findings reported were: sterile pyuria in 80(20%) patients, nausea and vomiting in 96(24%), abdominal pain in 85(21.3%), gallbladder distention in 14 (3.5%), transaminase elevation in 120 (30%), jaundice in 21(5.1%), irritability in 118(29.5%), aseptic meningitis in 16(4%), sensorineural hearing loss in 2 patients, uveitis in 11(2.7%) and arthritis or arthralgia in 55(13.8%). Cardiologic findings were: perivascular brightness of the coronary wall in 42(10.5%) patients, pericarditis in 9(2.3%), myocarditis in 4(1%), mitral regurgitation in 28(7%) and CA in 53 patients(13.3%), 26(49%) of them disappearing before the 2^nd^ month after the onset of KD. 4 patient had giant CA. Intravenous immunoglobulin (IVIG) was administered in 389(97.5%) patients with response to the 1^st^ dose in 332(83.2%). Day of IVIG administration was 7.5±3.1. Other treatment plans were: 2^nd^ (69% response) and 3^rd^ IVIG doses, oral or iv corticosteroids and abciximab (administered in 3 of the patients with giant CA). 97.7% of patients received anti-platelet dose aspirin in the convalescent phase.

## Conclusion

This is the first population-based study on the epidemiology of KD in Catalonia (Spain). It seems to be a higher incidence of CA in our cohort despite high rates of treatment response. Further analysis is required. Incidence rates, other clinical features and treatment plans are similar to dose described in studies in other European countries.

## Disclosure of interest

None declared.

